# Serotonin- and Dopamine-Related Gene Expression in* db/db* Mice Islets and in MIN6 *β*-Cells Treated with Palmitate and Oleate

**DOI:** 10.1155/2016/3793781

**Published:** 2016-06-05

**Authors:** L. R. Cataldo, M. L. Mizgier, D. Busso, P. Olmos, J. E. Galgani, R. Valenzuela, D. Mezzano, E. Aranda, V. A. Cortés, J. L. Santos

**Affiliations:** ^1^Departamento de Nutrición, Diabetes y Metabolismo, Escuela de Medicina, Pontificia Universidad Católica de Chile, 8331150 Santiago, Chile; ^2^Facultad de Medicina, Universidad de los Andes, 7620001 Santiago, Chile; ^3^UDA-Ciencias de la Salud, Carrera de Nutrición y Dietética, Escuela de Medicina, Pontificia Universidad Católica de Chile, 8331150 Santiago, Chile; ^4^Departamento de Nutrición, Facultad de Medicina, Universidad de Chile, 7550367 Santiago, Chile; ^5^Laboratorio de Hemostasia, Escuela de Medicina, Pontificia Universidad Católica de Chile, 8331150 Santiago, Chile

## Abstract

High circulating nonesterified fatty acids (NEFAs) concentration, often reported in diabetes, leads to impaired glucose-stimulated insulin secretion (GSIS) through not yet well-defined mechanisms. Serotonin and dopamine might contribute to NEFA-dependent *β*-cell dysfunction, since extracellular signal of these monoamines decreases GSIS. Moreover, palmitate-treated *β*-cells may enhance the expression of the serotonin receptor Htr2c, affecting insulin secretion. Additionally, the expression of monoamine-oxidase type B (Maob) seems to be lower in islets from humans and mice with diabetes compared to nondiabetic islets, which may lead to increased monoamine concentrations. We assessed the expression of serotonin- and dopamine-related genes in islets from* db/db* and wild-type (WT) mice. In addition, the effect of palmitate and oleate on the expression of such genes, 5HT content, and GSIS in MIN6 *β*-cell was determined. Lower Maob expression was found in islets from* db/db* versus WT mice and in MIN6 *β*-cells in response to palmitate and oleate treatment compared to vehicle. Reduced 5HT content and impaired GSIS in response to palmitate (−25%; *p* < 0.0001) and oleate (−43%; *p* < 0.0001) were detected in MIN6 *β*-cells. In conclusion, known defects of GSIS in islets from* db/db* mice and MIN6 *β*-cells treated with NEFAs are accompanied by reduced Maob expression and reduced 5HT content.

## 1. Introduction

Both insulin resistance and *β*-cell failure are involved in the etiology of type-2 diabetes (DM2) phenotype [1]. High circulating nonesterified fatty acids (NEFAs) concentration is often reported in fasting physiological state [2] or physiopathological conditions such as stressed states [3] and DM2 [4–6]. Lipid infusion in nondiabetic subjects leading to circulating NEFAs levels similar to those found in diabetic people (500–800 *μ*M) decreased glucose-stimulated insulin secretion (GSIS) [7]. Similar results were obtained in animal models [8]. Moreover, several* in vitro* studies have demonstrated that long-term exposure of *β*-cells lines and murine islets to palmitic (C16:0) and oleic (C18:1) acids leads to impaired GSIS [5, 8–10]. In rat pancreatic islets, the mechanism of *β*-cell lipodysfunction has been associated with reduced glucose uptake and mitochondrial oxidation caused by decreased activity of pyruvate dehydrogenase (PDH) and increased expression of UCP2 [11]. Other studies have explained impaired GSIS as a consequence of excessive production of radical oxygen species (ROS), which is dependent on mitochondrial fatty acid metabolism [9]. However, these mechanisms do not fully explain such decreased GSIS, and alternative mechanisms may be taking place.

Other molecular signals including serotonin and dopamine might contribute to NEFA-dependent *β*-cell dysfunction. In this context, high circulating levels of serotonin are observed under fasting conditions [2] and also in patients with DM2 [12, 13]. Furthermore, dietary fat composition may alter serotonin and dopamine concentration in the brain [14]. Moreover, NEFAs may directly modify the expression of some monoamines signaling genes in *β*-cells. It has been reported that expression of the serotonin receptor Htr2c is higher in pancreatic islets from diabetic versus control mice, and it is positively regulated in mice *β*-cells exposed to palmitic acid [15]. Moreover, agonists of Htr2c reduce the GSIS and may contribute to NEFAs-dependent *β*-cell dysfunction [15]. In addition, it has been recently reported that the activity of monoamine-oxidase type B (Maob), which degrades the monoamines, is reduced in diabetic human and mice islets [16], suggesting that higher content of monoamines in *β*-cells contributes to reduced GSIS in DM2. In support of this hypothesis, gut-derived serotonin and dopamine [2, 17–19] or synthesized [18, 20] and coreleased from *β*-cells could, in an endocrine or auto/paracrine manner, negatively modulate GSIS [15, 18, 19, 21–27]. In fact, serotonin acting through Htr2c receptor and dopamine through D2 or D3 receptor decrease GSIS in *β*-cell lines and human or mice islets [27–31]. In addition, antagonism of dopamine D2 receptors signaling in human islets enhanced GSIS [27].

In this regard, long-term exposure of *β*-cells to NEFAs may change the expression of some monoamines-related genes leading to increased serotonin and dopamine content and chronic autocrine signaling that consequently impair GSIS. The aims of this study were (i) to assess and qualitatively compare the expression of serotonin- and dopamine-related genes in pancreatic islets from wild-type (WT) and* db/db* mice and (ii) to evaluate the effect of palmitate and oleate on expression of such genes, 5HT content, and GSIS in MIN6 *β*-cells.

## 2. Material and Methods

### 2.1. MIN6 Cell Culture

MIN6 *β*-cells were cultured in Dulbecco's modified Eagle medium (DMEM) (Gibco; Life Technologies Co., Grand Island, NY) containing 10% fetal bovine serum (FBS), 25 mmol/L glucose, 3.7 g/L sodium bicarbonate, 100 U/mL penicillin, and 100 *μ*g/mL streptomycin. MIN6 *β*-cells were cultured at 37°C in a humidified atmosphere containing 95% air and 5% CO_2_. These cells were provided by Professor Francisco Pérez-Bravo (University of Chile, Santiago, Chile).

### 2.2. NEFAs Preparation and Treatment Protocol

Sodium salts solutions of nonesterified palmitic (Sigma, code P9767) and oleic (Sigma, code O7501) acids were prepared as previously reported [34]. Briefly, 1 mL of 100 mM palmitic or oleic solutions (27.8 mg of palmitate and 30.4 mg oleate) was dissolved in NaOH 0.1 M and warmed up to 60°–70°C and gently shaken. Then, the solutions were diluted twenty times in DMEM medium with 10% fatty acid-free Bovine Serum Albumin (BSA). Diluted NEFAs solutions were filtered (0.45 *μ*m), aliquoted in amber tubes, and frozen at −20°C. Experiments were then conducted by diluting 10x with fresh and sterile DMEM medium (10% fetal bovine serum, FBS) to reach the final concentration of 0.5 mM of NEFAs salts and 1% BSA.

MIN6 *β*-cells were incubated with palmitate or oleate (0.5 mM, 1% BSA) in DMEM medium (10% FBS) for 24 hours, and then glucose-stimulated insulin secretion was assessed. In parallel, the viability of MIN6 *β*-cells after 24 h of exposure to fatty acids was measured by Trypan Blue exclusion assay and the percentage of viability was quantified by an automated cell counter (Luna, Logos Biosystems).

### 2.3. Glucose-Stimulated Insulin Secretion (GSIS) Assay

MIN6 *β*-cells were evaluated by means of the static GSIS protocols. Briefly, GSIS protocol consisted in sensitizing the MIN6 *β*-cells by exposing them to Krebs-Ringer HEPES buffer (KRH; NaCl 130 mmol/L, KH_2_PO_4_ 1.25 mmol/L, MgSO_4_ 1.25 mmol/L, CaCl_2_ 2.68 mmol/L, NaHCO_3_ 5.26 mmol/L, and HEPES 10 mmol/L) without or with low glucose (2.8 mM), during thirty minutes. Immediately after that, MIN6 *β*-cells were stimulated to secrete insulin with KRH buffer (0.5% BSA) without or with glucose (20 mM) for one hour. The supernatants were spun to 5000 rpm during 10 minutes to 4°C and storage to −20°C. Later, the extracellular (secreted) insulin concentration was measured using the mouse/rat insulin ELISA kits (Merck-Millipore, code EZRMI-13K). GSIS was expressed as either the concentration of insulin secreted, at basal and stimulated glucose levels (normalized by total mass protein), or through the ratio of both, defined as the Stimulation Index (SI).

### 2.4. Animal Models

*db/db* mice were obtained from Jackson Laboratories (strain B6.BKS(D)-Leprdb/J). Male, 16–20-week-old wild-type (WT) C57BL6/J (WT (24–30 g) and* db/db* (55–60 g)) mice were studied.

This research was conducted in accordance with the experimental protocol approved by the Bioethical and Animal Welfare Committee from the School of Medicine, Pontificia Universidad Católica de Chile.

### 2.5. Plasma Insulin and NEFAs Assessment

Insulin and total NEFAs measurements were performed from a pool of plasma of* db/db* and WT mice (*n* = 5 per group). The total NEFAs quantitation was performed by the colorimetric test NEFA HR (Wako, code 993-35191) and the insulin levels by ELISA (Merck, code EZRMI-13K).

To quantify the specific NEFAs species from plasma pools, total lipids were quantitatively extracted by Bligh and Dyer method [35]. Briefly, the samples were homogenized with ice-cold chloroform/methanol (2 : 1 v/v) containing 0.01% butylated hydroxytoluene (BHT) as an antioxidant. Then the fatty acid methyl ester (FAME) were prepared and processed by Bond Elut NH_2_ columns (Agilent Technologies, USA) and the NEFAs were eluted with hexane. The NEFAs were separated and quantified by gas-liquid chromatography in Agilent Hewlett-Packard equipment (model 7890A, CA, USA) using a capillary column (Agilent HP-88, 100 m × 0.250 mm; ID 0.25 *μ*m) as previously reported [36].

### 2.6. Pancreatic Islets Isolation

Pancreatic islets isolation from mice was carried out based on standard, previously reported protocols, with some modifications [37, 38]. Briefly, mice were anesthetized by an intraperitoneal injection of ketamine/xylazine (100/10 mg/kg) before surgery. The pancreases were removed by laparotomy after perfusion with 3 mL of collagenase (0.21 mg/mL, Liberase TL Research Grade, Roche; code: 5401020001) through the common bile duct. Pools of three pancreases per tube were incubated during 14 min at 37°C, and the digestion reaction was stopped with RPMI medium plus 10% FBS. After several washes and centrifugations (200 g, 2 min, 4°C) homogenates were filtered using a 250 *μ*m wire mesh, and islets were separated by Histopaque 1077 (Sigma, cat. 10771) gradient. Isolated islets were rapidly stored in lysis buffer (Ambion PureLink System, Life Technologies) at −80°C until the pool of islets was gathered completely.

### 2.7. Gene Expression Analysis

**Figure 1:**
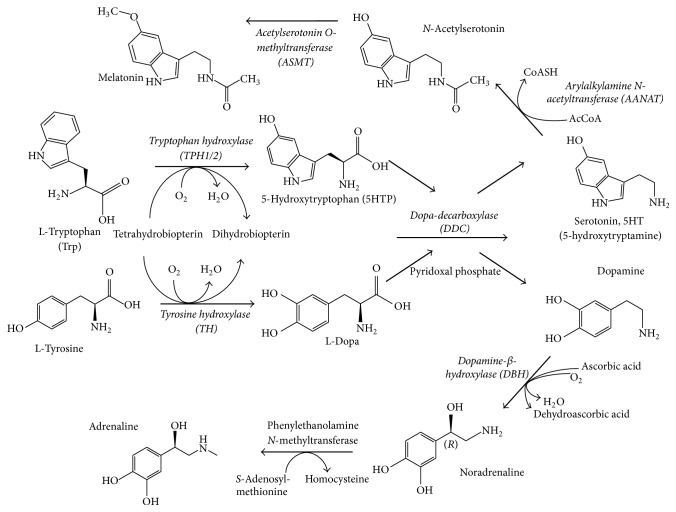
Diagram of biosynthetic pathways of monoamines. Serotonin (5-hydroxytryptamine or 5HT) biosynthesis is carried out by two sequential steps: (1) conversion of the essential amino acid tryptophan into 5-hydroxytryptophan (5HTP) by the enzyme tryptophan hydroxylase (TPH) and (2) decarboxylation of 5HTP to serotonin catalyzed by the enzyme Dopa-decarboxylase (DDC). Two isoforms of tryptophan hydroxylase are known: TPH1, which is found in nonneurons cells, and TPH2, which is predominantly found in neurons cells (mainly in CNS) [32], being both subtypes expressed in pancreatic *β*-cells [33]. Two additional enzymatic steps from serotonin are involved in melatonin synthesis, while DDC is also involved in the synthesis of dopamine from L-Dopa, which in turn is synthetized from the amino acid tyrosine by the action of enzyme tyrosine hydroxylase (TH). Finally, noradrenaline and adrenaline are synthetized from dopamine through the consecutively action of dopamine *β*-hydroxylase (DBH) and phenylethanolamine* N*-methyltransferase. The enzymes whose gene has been evaluated for expression were highlighted in italic.

Expression of several genes related to metabolism, degradation, and transport of serotonin and dopamine, as well as some genes related to synthesis of melatonin (synthetized from serotonin) and adrenaline/noradrenaline (from dopamine), was included in this study (Figure 1 and Table 2). Additionally, typical genes related to pancreatic *β*-cell function were evaluated (Table 1). For mRNA quantification, total RNA was isolated from MIN6 *β*-cells or from a pool of 335 and 312 islets from WT (*n* = 11) and* db/db* (*n* = 12) male mice, respectively, using the affinity columns systems (Ambion PureLink System, Life Technologies, code 12183018A). The mRNA was transformed in cDNA using reverse transcriptase (RT2 first-strand kit, code 330401, Qiagen), which was analyzed in the Stratagene Mx3000P equipment using RT-Profiler Custom PCR-Array (Custom Profiler, code CAPM12071 RT2, SABiosciences). Gene expression profiles were determined by PCR-Array platform that allows for simultaneous quantification of mRNA expression of several genes in a single PCR reaction. The gene expression profiles of NEFAs-treated and control MIN6 *β*-cells were quantified in three independent experiments, while only one determination was run for each pool of pancreatic islets from* db/db* and WT mice. The mRNA levels are expressed as fold change in 2^−ΔCt^ (Ct) (delta Ct means the Ct of the target gene minus the average Ct of three housekeeping genes: *β*-actin, glyceraldehyde-3-phosphate dehydrogenase, and *β*-glucuronidase) of a specific gene in the treated versus control conditions. For experiments involving pancreatic islets, the mRNA levels are expressed as fold change of 2^−ΔCt^ of target genes in islets from* db/db* relative to WT mice. In this case, the changes in gene expression were qualitatively compared and, due to the absence of statistical analysis, changes of biological relevance were arbitrarily defined as fold change higher than 2.0 or lower than 0.5 of genes with basal expression levels of at least Ct = 32.

### 2.8. Intracellular 5HT Analysis

MIN6 *β*-cells were supplemented with or without the 5HT precursor 5HTP (50 *μ*M) and treated with palmitate (0.5 mM), oleate (0.5 mM), or the Mao inhibitor Pargyline (20 *μ*M) during twenty-four hours. After treatments, cells were washed with PBS, trypsinized, collected in tubes, and centrifuged 200 g for 5 minutes. The cells pellets were resuspended in 500 *μ*L of 0.5 M perchloric acid (PCA) plus 50 *μ*L of internal standard and rapidly frozen to −80°C for 5 minutes. Then, cells were resuspended, sonicated for 10 minutes, homogenized by vortexing, and left on ice for 10 minutes for complete precipitation of proteins. Samples were then centrifuged at 16.000 g for 20 minutes at 4°C. The supernatant samples were stored at −80°C until 5HT analysis. The protein pellets were resuspended in 100 *μ*L of 1.0 N NaOH for protein quantification.

HPLC-electrochemical determination of intracellular 5HT was based on previously reported technical studies [39]. Briefly, 20 *μ*L of filtered supernatant samples was injected into an HPLC system with the following configuration: a dC18 of 3 *μ*m column (Atlantis), an isocratic pump (Waters E2695), and an amperometric detector (set at 500 mV, 10 nA, and latency of 5 seconds) using the software EMPOWER. The mobile phase containing 0.1 M sodium acetate, 0.1 M citric acid, 1.0 mM EDTA, and 12% methanol (pH adjusted to 4.6) was pumped at a flow rate of 1 mL/min. The retention time for 5HT was 5.3 min and for internal standard (*N*-methyl-5-hydroxytryptamine oxalate) was 6.0 min.

### 2.9. Statistical Analysis

Data are expressed as mean ± standard errors of measurements of at least three independent experiments. Student's *t*-tests or one-way ANOVA tests were carried out depending on whether comparisons involved two or more groups. Significant associations were declared at a nominal level of *p* = 0.05.

## 3. Results

### 3.1. Metabolic Alteration and mRNA Expression in Islets from* db/db* Mice

The* db/db* mice are an obesity-induced diabetes model [40, 41]. Concordantly,* db/db* compared to WT mice display obesity (55–60 g versus 24–30 g), hyperinsulinemia (11.3 versus 0.7 ng/mL), and high plasma levels of total NEFAs (0.93 versus 0.34 mM). Furthermore, it is known that* db/db* mice islets have functional defects and increased compensatory *β*-cell mass [40–42]; in agreement we observed a higher size in islets from* db/db* compared to WT mice (data not shown).

To further study the metabolic disturbance, the mRNA expression levels of some genes related to glucose, lipids, and mitochondrial metabolism were measured in islets from* db/db* and WT mice and they were compared qualitatively (Table 1). Islets from* db/db* versus WT mice showed lower expression of glucose transporter 2 gene Slc2a2 (Glut2) (0.33-fold; Table 1). In turn, lipid receptor genes such as Ffar2 and Gpr119 had increased (4.29-fold and 2.07-fold), while O3far1 decreased expression (0.10-fold) in islets from* db/db* compared with WT mice (Table 1). Mitochondrial-related genes including PGC1*α* (0.46-fold) and ATP synthase alpha subunit (Atp4a) (0.34-fold) showed lower expression while a higher expression was observed for uncoupled protein 2 (UCP2) (2.06-fold) and subunit of the mitochondrial complex I (Ndufa1) (2.13-fold) in islets from* db/db* versus WT mice (Table 1).

### 3.2. mRNA Expression of Serotonin- and Dopamine-Related Genes in Islets from* db/db* Mice

Most of the genes involved in monoamine biosynthesis (Tph1/2, Ddc, Th, and Dbh), vesicular transporters (Slc18a1/2-VMAT1/2), and degradative enzymes (Maoa/b and Comt) were expressed in WT islets. The most highly expressed gene was Ddc (Ct = 22.7) that encode the metabolic crossroad enzyme of serotonin and dopamine synthesis (DDC). The second most highly expressed gene was the monoamine-oxidase type (Maob) (Ct = 23.4), encoding the enzyme that degrades monoamines (Maob) (Table 2).

When the expression of these genes was qualitatively compared among islets from* db/db* and WT mice, Tph2 (2.1-fold), Ddc (2.0-fold), Aanat (3.1-fold), Slc18a1 (2.0-fold), and Slc18a2 (2.9-fold) showed higher expression, while Maob (0.37-fold) showed lower expression. The serotonin and dopamine plasma membrane transporter genes (slc6a4-SERT, slc6a3-DAT) were undetectable in islets from* db/db* mice (Table 2).

### 3.3. Glucose-Stimulated Insulin Secretion in NEFAs-Treated MIN6 *β*-Cells

**Figure 2:**
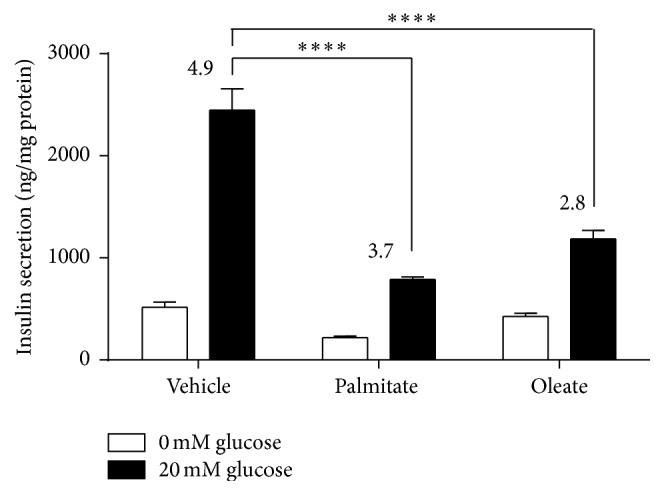
Glucose-stimulated insulin secretion in NEFAs-treated MIN6 *β*-cells. MIN6 *β*-cells were treated during twenty-four hours with palmitate or oleate (0.5 mM) and then GSIS was evaluated with KRH buffer with or without glucose (20 mM) during one hour. Graph bars represent the insulin secretion at low (white bars) or high glucose (black bars). Graph bars represent mean ± standard errors of measurements of three independent experiments in triplicate. The numbers above bars represent the Stimulation Index (ratio of insulin secreted in cells with and without glucose). The statistical analysis was made comparing treated versus control at basal and stimulated condition. The symbol *∗∗∗* *∗* denotes *p* < 0.0001 relative to vehicle condition in one-way ANOVA.

Total lipids from a pool of plasma of* db/db* and WT mice (*n* = 5 per group) were quantitatively analyzed and confirmed that the most abundant specific NEFAs were the saturated palmitic acid (C16:0) and the monounsaturated oleic acid (C18:1) (data not shown). Thus, to ascertain whether high levels of these NEFAs directly impair *β*-cell function, the glucose-stimulated insulin secretion (GSIS) was evaluated in MIN6 *β*-cells after long-term treatment with palmitate and oleate (24 hours). To avoid potentially confounding influences in gene expression related to cytotoxic effect of chronic NEFAs exposure, the viability was measured, confirming no significant decrease in cell viability after NEFAs treatment (data not shown). As shown in Figure 2, the control cells treated with vehicle were highly responsive to glucose, increasing a mean of 5-fold the insulin secretion after glucose stimulation (20 mM) compared to basal release (without glucose) (Stimulation Index, SI) (Figure 2). When MIN6 *β*-cells were exposed to palmitate and oleate, the GSIS decreased by 25% and 43% relative to vehicle condition, respectively (SI = 3.7 ± 0.2; *p* < 0.0001 and 2.8 ± 0.1; *p* < 0.0001, Figure 2).

### 3.4. mRNA Expression of Energy Metabolism Genes in NEFAs-Treated MIN6 *β*-Cells

**Figure 3:**
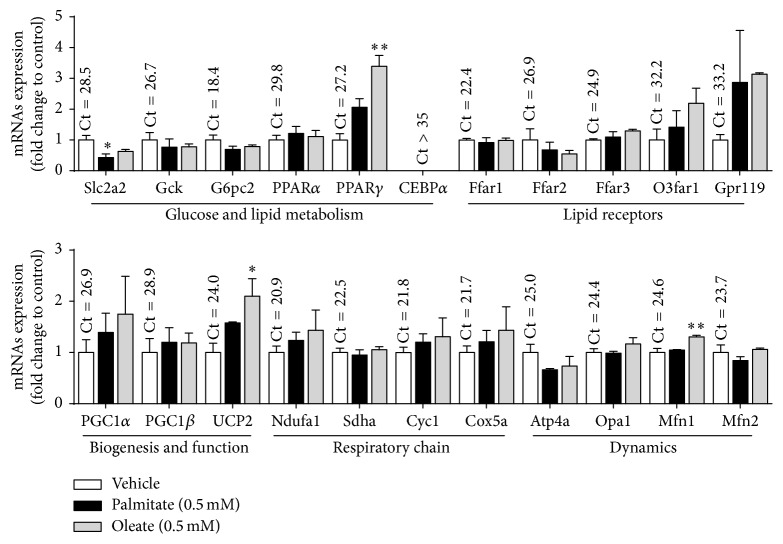
mRNA expression of energy metabolism-related genes in NEFAs-treated MIN6 *β*-cells. MIN6 *β*-cells were treated during twenty-four hours with palmitate or oleate (0.5 mM). Then, total RNA was extracted and the mRNA levels of genes related to glucose and lipid metabolism and with mitochondrial function were evaluated by RT-qPCR. The mRNA levels are expressed as fold change in 2^−ΔCt^ of the specific gene in the treated versus control conditions (see Section 2). Graph bars represent mean ± standard errors of measurements of three independent experiments in triplicate. The numbers above bars represent the mean Ct value in control cells. The symbol *∗* denotes *p* < 0.05 and *∗∗* denotes *p* < 0.01 in one-way ANOVA.

In order to understand the GSIS impairments, the mRNAs levels of some genes related to glucose, lipid, and mitochondrial metabolism in MIN6 *β*-cells exposed to palmitate and oleate were evaluated (Figure 3).

Among the glucose metabolism-related genes, the expression of glucose transporter gene Slc2a2 (Glut2) was decreased in MIN6 *β*-cells in response to palmitate but not with oleate treatment compared to vehicle (0.48 ± 0.12; *p* = 0.049 and 0.63 ± 0.062; *p* = 0.168) (Figure 3). Glucokinase (Gck) and glucose 6 phosphatase (G6pc2) showed no significant changes in response to NEFAs treatment (Figure 3). Among lipid-related transcription factor genes only PPAR*γ* was upregulated after NEFAs treatment, being statistically significant only with oleate treatment (2.87 ± 0.56; *p* = 0.027). The fatty acids receptor genes Ffar1 (Gpr40), Ffar2 (Gpr43), and Ffar3 (Gpr41) were highly expressed and O3far1 (Gpr120) and Gpr119 were poorly expressed in MIN6 *β*-cells at basal conditions, while their differences did not reach a statistical significance after NEFAs treatment (Figure 3). Among genes related to mitochondrial biogenesis, function, and dynamics, gene encoded uncoupled protein 2 (UCP2) was increased in MIN6 *β*-cells in response to palmitate and oleate but only reaching statistically significance with oleate (2.10 ± 0.34; *p* = 0.019). The gene of mitofusin 1 (Mfn1) was also increased after oleate treatment (1.31 ± 0.03; *p* = 0.009) (Figure 3).

### 3.5. mRNA Expression of Monoamines-Related Genes in NEFAs-Treated MIN6 *β*-Cells

**Figure 4:**
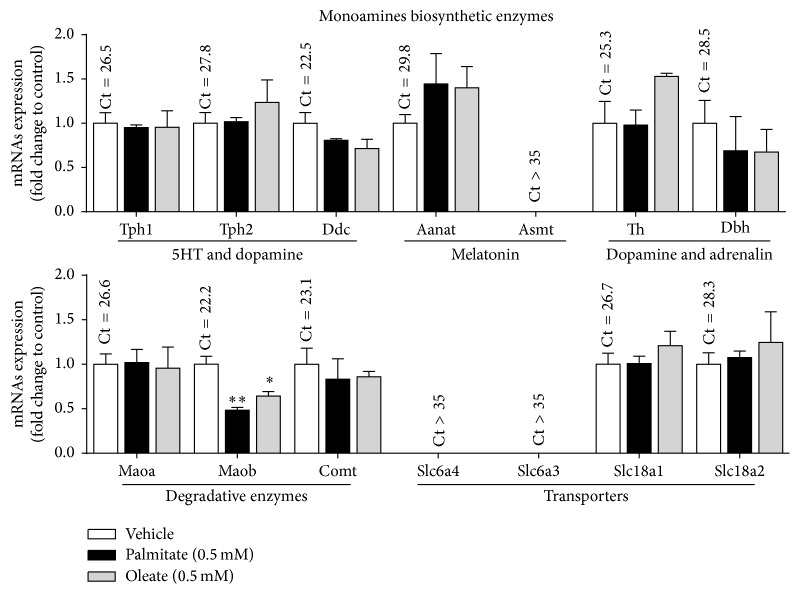
mRNA expression of monoamines-related genes in NEFAs-treated MIN6 *β*-cells. MIN6 *β*-cells were treated during twenty-four hours with palmitate or oleate (0.5 mM). Then, total RNA was extracted and mRNA levels of genes related to the monoamines biosynthesis, degradation, and transport were evaluated by RT-qPCR. The mRNA levels are expressed as fold change between 2^−ΔCt^ of the specific gene in the treated versus control conditions (see Section 2). Graph bars represent mean ± standard errors of measurements of three independent experiments in triplicate. The numbers above bars represent the mean Ct value in control cells. The symbol *∗* denotes *p* < 0.05 and *∗∗* denotes *p* < 0.01 in one-way ANOVA.

The mRNA expression levels of several genes related to biosynthesis and metabolism of monoamines were quantified in MIN6 *β*-cells. As shown in Figure 4, MIN6 *β*-cells at basal condition expressed almost all genes encoding enzymes needed for serotonin (Tph1/2 and Ddc), dopamine (TH and Ddc), and noradrenalin (TH, Ddc, and Dbh) synthesis (Figure 1). The most highly expressed gene was Maob (Ct = 22.2), while the second most highly expressed was Ddc (Ct = 22.5). Similar to mice islets, no expression of the main plasma membrane transporter genes of serotonin (slc6a4-SERT) and dopamine (slc6a3-DAT) (Ct > 35) was detected in MIN6 *β*-cells.

Then, the expression changes of these genes in MIN6 *β*-cells in response to palmitate and oleate treatment were evaluated. None of these genes showed significant expression changes in MIN6 *β*-cells with exception of the Maob, which significantly decreased in response to palmitate and oleate compared to control (0.48 ± 0.03; *p* = 0.002 and 0.64 ± 0.05; *p* = 0.014, resp.) (Figure 4).

### 3.6. Intracellular 5HT Content in NEFAs-Treated MIN6 *β*-Cells

**Figure 5:**
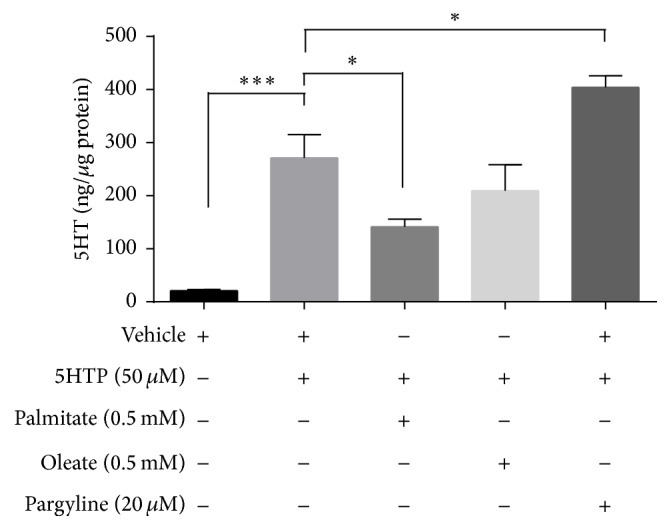
Intracellular 5HT content in NEFAs-treated MIN6 *β*-cells. MIN6 *β*-cells were supplemented with or without the 5HT precursor 5HTP (50 *μ*M) and treated with palmitate (0.5 mM), oleate (0.5 mM), or the Mao inhibitor Pargyline (20 *μ*M) during twenty-four hours. Then, cells were collected and homogenized and the protein was separated by PCA precipitation. The 5HT concentration was quantified by HPLC in supernatant and adjusted to total protein content (see Section 2). Graph bars represent mean ± standard errors of measurements of three independent experiments in duplicate. The symbol *∗* denotes *p* < 0.05 and *∗∗∗* denotes *p* < 0.001 in one-way ANOVA.

It was assessed whether the treatment of MIN6 *β*-cells with palmitate and oleate may change intracellular 5HT content. In control conditions, the 5HT content in MIN6 *β*-cells was detected in low levels (Figure 5). However, such level strongly increased after supplementation with the 5HT precursor 5HTP, confirming the existence of a functional microserotonergic system in *β*-cells. When the 5HTP supplemented MIN6 *β*-cells were additionally treated with palmitate, a significant reduction of intracellular 5HT content was observed (271.6 versus 141.5 ng of 5HT/*μ*g protein; *p* = 0.0479); similar trend was observed with oleate. As expected, in MIN6 *β*-cells supplemented with 5HTP and simultaneously treated with the Mao inhibitor Pargyline, the 5HT content was further increased compared with cells only incubated with 5HTP (Figure 5).

## 4. Discussion

**Figure 6:**
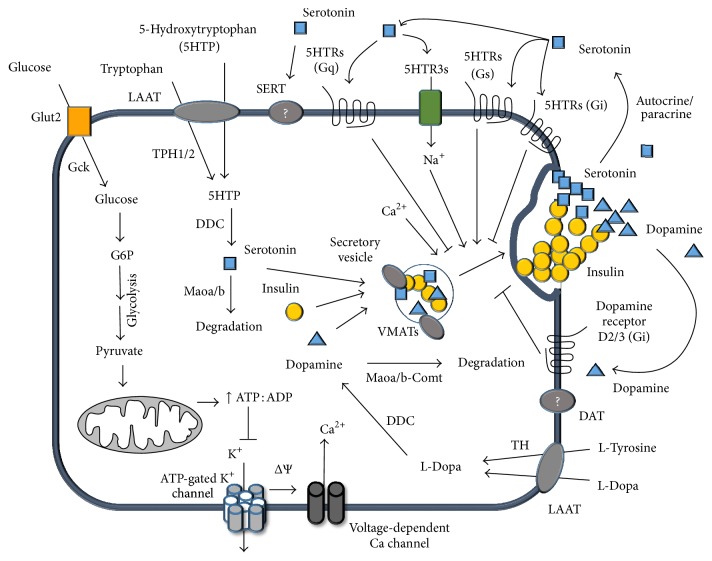
Outline of serotoninergic and dopaminergic systems in *β*-cells and its role in insulin secretion. Glucose enters into pancreatic *β*-cell via the insulin independent glucose transporter (Glut2 in mice), allowing its metabolism through glycolysis, Krebs cycle, and oxidative phosphorylation. The increase in the ATP/ADP ratio induces the inhibition of the ATP dependent potassium channel and consequently the plasma membrane depolarization, triggering the extracellular calcium uptake and the insulin granules exocytosis. Both serotonin precursors, 5HTP and L-tryptophan, and the dopamine precursors, L-tyrosine and L-Dopa, can enter into the *β*-cell by the LAAT transporter. The first two precursors may be converted to serotonin by action of the enzymes TPH1/2 and DDC, while the latter precursors could be converted to dopamine by the enzymes TH and DDC. These newly synthesized monoamines are stored in the secretory granules by the vesicular carriers (VMAT1/2). In response to glucose stimuli, the *β*-cells can release insulin, together with serotonin and dopamine. Therefore, both extracellular monoamines can exert their actions on insulin secretion depending on which specific receptors are activated. Serotonin and dopamine may inhibit the glucose-stimulated insulin secretion acting by Htr2c and D3 receptors, respectively. We propose that the exposure of pancreatic *β*-cells to the nonesterified fatty acids, palmitic and oleic acids, may lead to a decreased expression of Maob and thus an accumulation of the monoamines, serotonin and dopamine. When these monoamines are released, they signal through their specific receptors, which may contribute to a decreased glucose-stimulated insulin secretion.

In this work, we have shown that islets from WT and* db/db* mice expressed mRNAs of most genes related to the serotonin and dopamine metabolism, including enzymes involved in the synthesis (Tph1/2, TH, and Ddc), vesicular transport (Slc18a1/2-VMAT1/2), and degradation (Maoa, Maob, and Comt) of these monoamines. Conversely, the genes of the main cell membrane transporter of these monoamines (slc6a4-SERT, slc6a3-DAT) were almost undetectable (Figure 6). The* db/db* mice are an animal model of diabetes characterized by high plasma levels of NEFAs and insulin secretory defects of *β*-cells [40–42]. Remarkably, islets from* db/db* compared to WT mice had higher mRNA levels of the serotonin and dopamine common synthesis enzyme (Ddc) and vesicular transporters VMAT1/2 and lower mRNA level of Maob, the degradative enzyme of the monoamines. These differences in gene expression suggest that islets from* db/db* mice have an increased synthesis and storage and decreased degradation, which might enhance autocrine signaling of serotonin and dopamine in *β*-cells (Figure 6). However, it is not possible to draw definitive conclusions of mRNA expression changes obtained in our study because these were carried out in pools of islets from* db/db* and WT mice rather than in independent experiments. This weakness is related to the difficulty to obtain islets from the scarce* db/db* male mice.

Similarly to islets from WT mice, MIN6 *β*-cells at basal conditions (vehicle) express most of the mRNAs of serotonin and dopamine metabolism genes mentioned above and cell membrane transporter genes (slc6a4-SERT, slc6a3-DAT) were undetectable. However, this does not rule out the possibility that *β*-cells can take up monoamines because other less known serotonin and dopamine transporters, such as the plasma membrane monoamines transporter (PMAT) and organic cation transporter 3 (OCT3) [43], could be expressed. In fact, gene expression databases indicate that PMAT and OCT3 are present in both human and mice islets and in different *β*-cell lines (https://www.t1dbase.org/page/AtlasView).

As expected, palmitic and oleic acids were the most abundant, saturated, and monounsaturated NEFAs in plasma of mice, with higher circulating concentration found in plasma of* db/db* mice compared to WT mice. In agreement with other studies [5, 6, 9, 44], here we show a decreased GSIS in MIN6 *β*-cells treated with palmitate and oleate versus vehicle. Considering previous reports of an inverse correlation between the fetal bovine serum concentration and cytotoxic action of NEFAs [45, 46], we used a mild protocol and discarded the influence of *β*-cell death on the observed decreased GSIS.

The gene expression changes observed in MIN6 *β*-cells in response to NEFAs treatment were compatible with a decreased use of glucose as the energy source that explains the reduced GSIS. In this regard, we found a decrease in the mRNA of glucose transporter Glut2 and an increase in UCP2 and PPAR*γ* in MIN6 *β*-cells treated with palmitate or oleate versus vehicle, respectively. Importantly, we have observed that both genes, Glut2 and UCP2, regulated by NEFAs in MIN6 *β*-cells, are correspondingly different in islets from* db/db* versus WT mice. The expression changes of such genes have been associated with decreased GSIS [47–49]. A decreased Glut2 transporter implies a reduced glucose uptake and oxidation [50], while a higher UCP2 expression leads to decreased coupling of mitochondrial respiration and ATP production [51] and defective mitochondrial respiration is a mechanism that explains decreased GSIS on NEFAs long-term treated *β*-cells [9, 52].

Remarkably, we have found a lower Maob expression both in islets from* db/db* compared to WT mice and in MIN6 *β*-cells exposed to palmitate and oleate compared to vehicle. Concordantly with our findings, a recent study showed lower Maob protein expression in insulin producing cells of diabetic humans and mice compared to nondiabetic controls [16]. Some reports have previously shown that Maob expression and activity is limited to *α*- and *β*-cells of endocrine rather than other cells of entire pancreas of mice or rat [16, 53, 54].

Pharmacologic* in vivo* studies demonstrated that pretreatment of mice with either of two Mao inhibitors, Pargyline or Nialamide, decreased the insulin secretion stimulated by different agents [55]. This effect is consistent with the observation that inhibition of Mao triggered increased duration of dopamine signaling in the *β*-cell, together with a decreased insulin secretion [55].* In vitro* studies have shown a dual effect of Mao inhibition on insulin secretion in rat pancreatic islets. Treatment of them with Mao inhibitor, in low concentrations caused an enhancement, while in higher concentrations it caused a reduced glucose-stimulated insulin secretion [54]. The most recent* in vitro* study confirm that inhibition of Maob reduces GSIS in mouse islets [16]. On the other hand, indirect evidences supporting the role of Maob in the function of pancreatic *β*-cells come from human clinical studies, which demonstrated an association between the long-term uses of tricyclic antidepressants, which inhibit the Mao activity, with an increased risk of diabetes incidence [56].

The monoamine content in *β*-cells is regulated by the activity of Mao, which also possess a selective substrate preference. In this sense, the major substrates to Maoa are tyramine, norepinephrine, epinephrine, and serotonin while Maob degrades preferably benzylamine, phenylethylamine, and dopamine. In this context, reduced Maob expression in *β*-cells may lead to higher content and signaling of different monoamines, especially dopamine, which has an inhibitory effect on GSIS in *β*-cells through D2 and D3 receptors [18, 19, 24, 27, 57] (Figure 6). Furthermore, it has been proposed dopamine as an anti-incretin hormone counteracting the positive action of glucagon-like peptide 1 (GLP1) on GSIS in *β*-cells [19].

Our results suggest that palmitate and oleate negatively regulate Maob expression on *β*-cells, possibly leading to increased dopamine content, secretion and subsequent signaling through dopamine receptors, which may contribute to decreased GSIS. To support this hypothesis, further studies should be performed to estimate dopamine content in islets from* db/db* and WT mice and in MIN6 *β*-cells exposed to NEFAs. On the other hand, it is unlikely that Maob reduced expression is related to 5HT content since this monoamine is preferentially degraded by Maoa, which has not changed in NEFAs-treated MIN6 *β*-cells. Concordantly, we have not found higher 5HT content levels in such treated cells and, conversely, 5HT content was even significantly reduced in MIN6 *β*-cells treated with palmitate. In this regard, although no significant changes were observed in expression levels of serotonergic genes in NEFAs-treated MIN6 *β*-cells, we cannot discard modifications in rates of 5HT synthesis, degradation, or release in response to NEFAs. Thus, the reduced 5HT content found in NEFAs-treated MIN6 *β*-cells could be explained by a higher 5HT secretion and signaling which in turn may contribute to decreased GSIS. Interestingly, radical oxygen species (ROS) are produced as byproducts of 5HT degradation that mediates oxidative stress and mitochondrial toxicity in different cell types [28]. Therefore, it is also possible that reduced 5HT content in NEFAs-treated MIN6 *β*-cells might be related to a putative compensation to reduce ROS derived from degradation of 5HT. Thus, future studies should be conducted to decipher which of these mechanisms may explain the decreased 5HT content in *β*-cells in response to NEFAs.

## 5. Conclusion

We confirmed that Maob expression was lower in islets from* db/db* versus wild-type mice and decreased in MIN6 *β*-cells exposed to palmitate and oleate compared to control, accompanied by decreased 5HT content and impaired GSIS. Our results suggest that impaired GSIS in *β*-cells in response to palmitate and oleate might be partly explained by decreased Maob expression, which in turn would lead to increased dopamine content, release, and signaling through specific receptors.

## Figures and Tables

**Table 1:** mRNA expression of energy metabolism-related genes in islets from *db/db* mice. Total RNA was extracted from a pool of 335 and 312 islets from WT (*n* = 11) and *db/db* (*n* = 12) male mice, respectively. Relative mRNA levels of genes related to glucose and lipid metabolism and to mitochondrial function were evaluated by RT-qPCR using custom RT-Profiler. The mRNA levels are expressed as fold-change in 2^−ΔCt^ of the specific gene in islets from *db/db* relative to WT mice (see Section 2).

Gene	Name	Islets Cts in		Fold change	Related pathway
		WT	*db/db*		
Slc2a2	Solute carrier family 2, member 2; Gut2	21.17	23.06	0.33	Glucose metabolism
Gck	Glucokinase	24.26	24.62	0.96	Glucose metabolism
G6pc2	Glucose-6-phosphatase, catalytic, 2	19.32	19.06	1.47	Glucose metabolism
PPAR*α*	Peroxisome proliferator activated receptor alpha	29.93	30.78	0.68	Lipids metabolism
PPAR*γ*	Peroxisome proliferator activated receptor gamma	26.14	26.86	0.75	Lipids metabolism
CEBP*α*	CCAAT/enhancer binding protein (C/EBP), alpha	27.79	29.66	0.34	Lipids metabolism
Ffar1	Free fatty acid receptor 1	24.17	24.58	0.93	Lipids receptors
Ffar2	Free fatty acid receptor 2	26.27	24.47	4.29	Lipids receptors
Ffar3	Free fatty acid receptor 3	26.45	26.49	1.20	Lipids receptors
O3far1	Omega-3 fatty acid receptor 1	28.51	32.08	0.10	Lipids receptors
Gpr119	G-protein coupled receptor 119	26.30	25.55	2.07	Lipids receptors
PGC1*α*	PPAR*γ* coactivator 1 alpha	30.00	30.66	0.46	Mitochondrial metabolism
PGC1*β*	PPAR*γ* coactivator 1 beta	27.80	29.21	0.78	Mitochondrial metabolism
UCP2	Uncoupling protein 2	21.33	20.59	2.06	Mitochondrial metabolism
Ndufa1	NADH dehydrogenase 1 alpha subcomplex, 1	21.89	21.10	2.13	Mitochondrial complex
Sdha	Succinate dehydrogenase complex, subunit A	22.59	23.29	0.76	Mitochondrial complex
Cyc1	Cytochrome c-1	23.84	23.84	1.23	Mitochondrial complex
Cox5a	Cytochrome c oxidase, subunit Va	23.79	24.61	0.70	Mitochondrial complex
Atp4a	ATPase, H+/K+ exchanging, alpha polypeptide	26.83	28.70	0.34	Mitochondrial dynamics
Opa1	Optic atrophy 1 homolog	25.86	25.64	1.43	Mitochondrial dynamics
Mfn1	Mitofusin 1	24.91	25.27	0.96	Mitochondrial dynamics
Mfn2	Mitofusin 2	24.26	24.7	0.91	Mitochondrial dynamics

**Table 2:** mRNA expression of serotonin- and dopamine-related genes in islets from *db/db* mice. Total RNA was extracted from a pool of 335 and 312 islets from WT (*n* = 11) and *db/db* (*n* = 12) male mice, respectively. Relative mRNA levels of genes related to the monoamines (MAs) biosynthesis, degradation, and transport were evaluated by RT-qPCR using custom RT-Profiler. The mRNA levels are expressed as fold-change in 2^−ΔCt^ of the specific gene in islets from *db/db* relative to WT mice (see Section 2).

Gene	Name	Islets Cts in		Fold change	Related pathway
		WT	*db/db*		
Tph1	Tryptophan hydroxylase 1	33.29	35	0.38	Serotonin biosynthetic pathway
Tph2	Tryptophan hydroxylase 2	31.04	30.27	2.10	Serotonin biosynthetic pathway
Ddc	Dopa decarboxylase	22.7	21.97	2.04	MAs biosynthetic pathway
Aanat	Arylalkylamine *N*-acetyltransferase	31.37	30.05	3.07	Melatonin biosynthetic pathway
Asmt	Acetylserotonin *O*-methyltransferase	>35	>35	Not expressed	Melatonin biosynthetic pathway
Th	Tyrosine hydroxylase	28.11	29.13	0.61	Adrenergic biosynthetic pathway
Dbh	Dopamine beta hydroxylase	33.94	35.00	0.59	Adrenergic biosynthetic pathway
Maoa	Monoamine oxidase A	28.33	28.94	0.81	MAs degradative enzymes
Maob	Monoamine oxidase B	23.43	25.17	0.37	MAs degradative enzymes
Comt	Catechol-*O*-methyltransferase	24.4	24.29	1.30	MAs degradative enzymes
Slc6a3	Solute carrier family 6, member 3	>35	>35	Not expressed	Dopamine transporter
Slc6a4	Solute carrier family 6, member 4	33.17	35.00	0.35	Serotonin transporter
Slc18a1	Solute carrier family 18, member 1; Vmat1	27.29	26.57	2.03	MAs transporters
Slc18a2	Solute carrier family 18, member 2; Vmat2	30.30	29.05	2.93	MAs transporters
